# A Rare Case of Severe Idiopathic Stuttering Priapism in a Young Healthy Man

**DOI:** 10.7759/cureus.2758

**Published:** 2018-06-07

**Authors:** Saeed Ali, Zeeshan Sattar, Sana Hussain, Farhan Sattar, Daniel Tambunan

**Affiliations:** 1 Internal Medicine Residency, Florida Hospital-Orlando, Orlando, USA; 2 Internal Medicine, Khyber Teaching Hospital, Peshawar, PAK; 3 Internal Medicine Residency, Khyber Teaching Hospital, Peshawar, PAK; 4 Internal Medicine, Ayub Teaching Hospital, Abbottabad, PAK; 5 Graduate Medical Education, Florida Hospital-Orlando, Orlando, USA

**Keywords:** priapism, stuttering, idiopathic

## Abstract

Priapism, a persistent erection of the penis which has no association with sexual activity and lasts longer than four hours, is a urologic emergency. It can be classified into ischemic, nonischemic, and stuttering categories. The pathophysiology of stuttering priapism is not well understood; however, the dysregulation of nitric oxide and phosophodiesterase-5 (PDE5) has been put forward as a possible mechanism. A 35-year-old male with a history of recurrent priapism presented with continuous penile erection for more than 48 hours. In the emergency room, penile aspiration and an intracavernous phenylephrine injection were attempted which did not help. Subsequently, a distal penile shunt was surgically created; however, the patient’s symptoms still persisted. A second round of penile irrigation, aspiration, and an intracavernous phenylephrine injection were attempted, but it was not helpful. Finally, another surgical shunt was created bilaterally between the corpora cavernosa and corpus spongiosum, which led to complete resolution of symptoms in the next 24 hours. The patient received an injection of lupron, and he was discharged.

## Introduction

Priapism is the term applied to a continuous erection of the penis unrelated to sexual stimulation [1]. More specifically, it lasts longer than four hours in duration [2]. It is among the most common urologic emergencies. The most frequent form is ischemic priapism which accounts for 95% of all episodes [3-4]. Stuttering priapism is a form of ischemic priapism which is recurrent in nature. It is characterized by multiple episodes of painful erections, and it is accompanied by periods of detumescence intermittently [2]. In 60% of these cases, the pathophysiology is not clear. The rest of the cases can be attributed to conditions like sickle cell disease, vasoactive injections, psychotropic medications, recreational drugs, and malignancy [5]. Complications of this condition include erectile dysfunction, penile pain, necrosis [6], and gangrene [7].

## Case presentation

**Figure 1:**
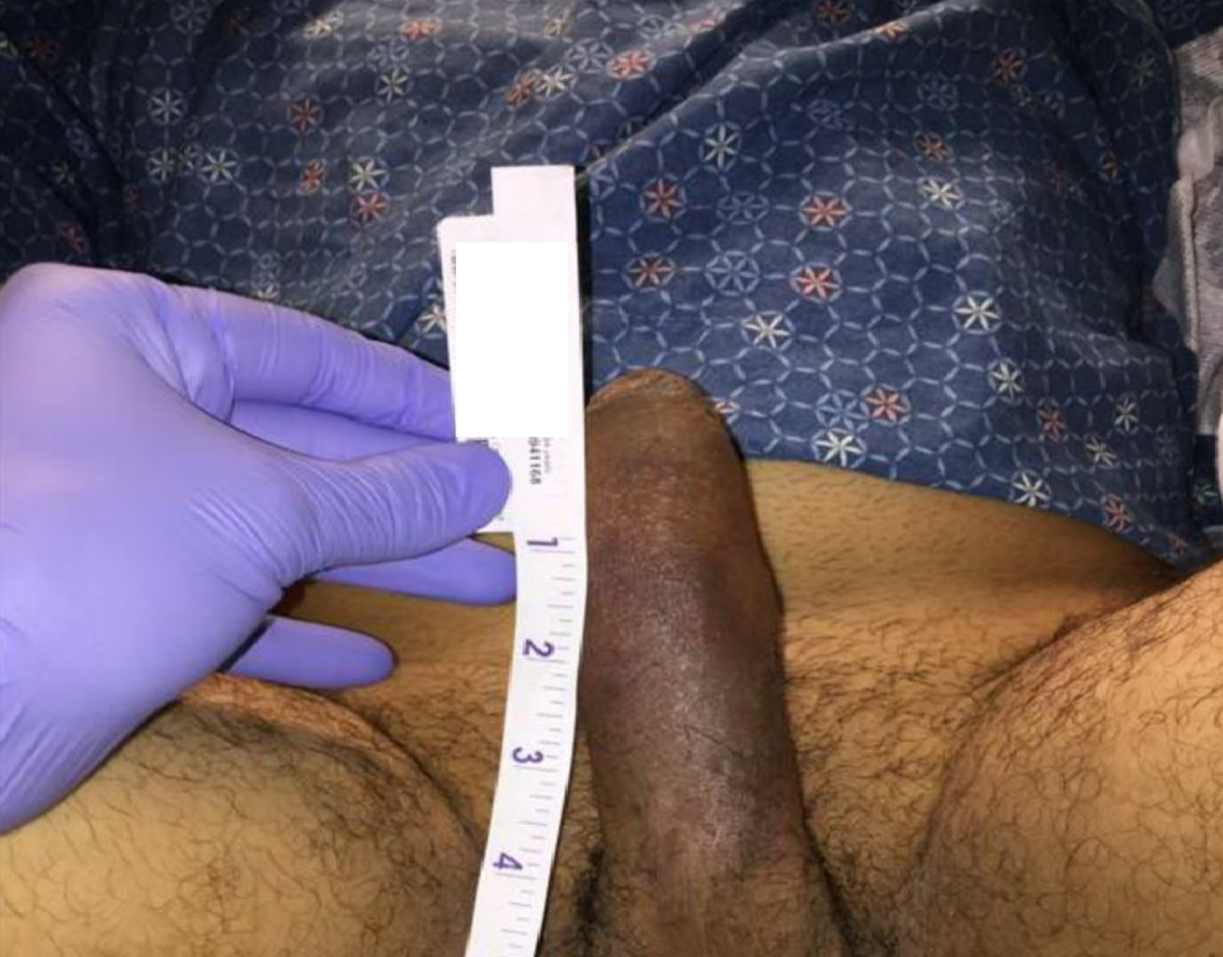
Erect penis after direct aspiration and an intracavernous phenylephrine injection.

A 35-year-old male with an unremarkable past medical history presented with a painful penile erection. He had woken up with a painful penile erection 48 hours ago which had persisted continuously since. He denied penile or perineal trauma, use of recreational drugs or medications, and personal or family history of sickle cell disease or other hematologic diseases. He had two similar episodes in the last six months. The first episode lasted for 24 hours and resolved spontaneously. The second episode lasted for more than 24 hours, and it required decompression with an intracavernous phenylephrine injection. On physical examination, he had an erect penis; however, the rest of the general and systemic examination was unremarkable. Initial lab tests revealed mild leukocytosis of 12 × 103/L, peripheral eosinophilia of 530 cells/L, and a normal hemoglobin level. Peripheral smear and reticulocyte counts were normal. Cavernous blood gas analysis showed paCO_2_ 103 mmHg, and paO_2_ < 5 mmHg, and pH 6.8. Lactate dehydrogenase (LDH) was mildly elevated at 294 U/L. Therefore, peripheral flow cytometry was obtained which was unremarkable. Urine drug screen was normal. Direct penile aspiration was attempted, which was not successful. The patient received an intracavernous phenylephrine injection, which did not help (Figure 1).

**Figure 2:**
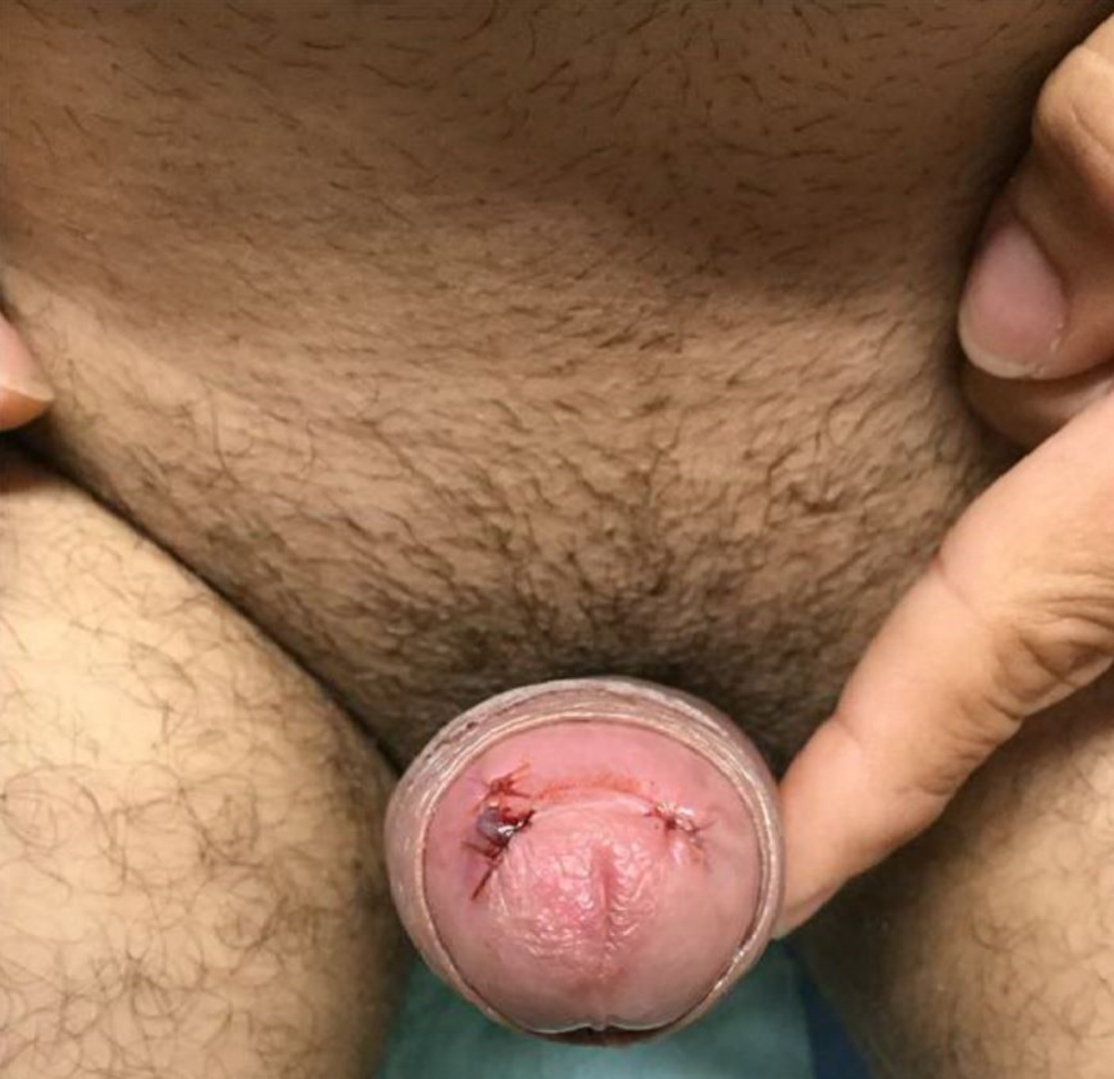
Persistent erection of the penis after distal penile shunt formation (Winter shunt).

The patient was taken to the operation room where penile irrigation was attempted followed by the formation of a distal penile shunt called Winter shunt. Postoperatively, the patient was observed till the next morning; however, his priapism did not resolve completely (Figure 2).

**Figure 3:**
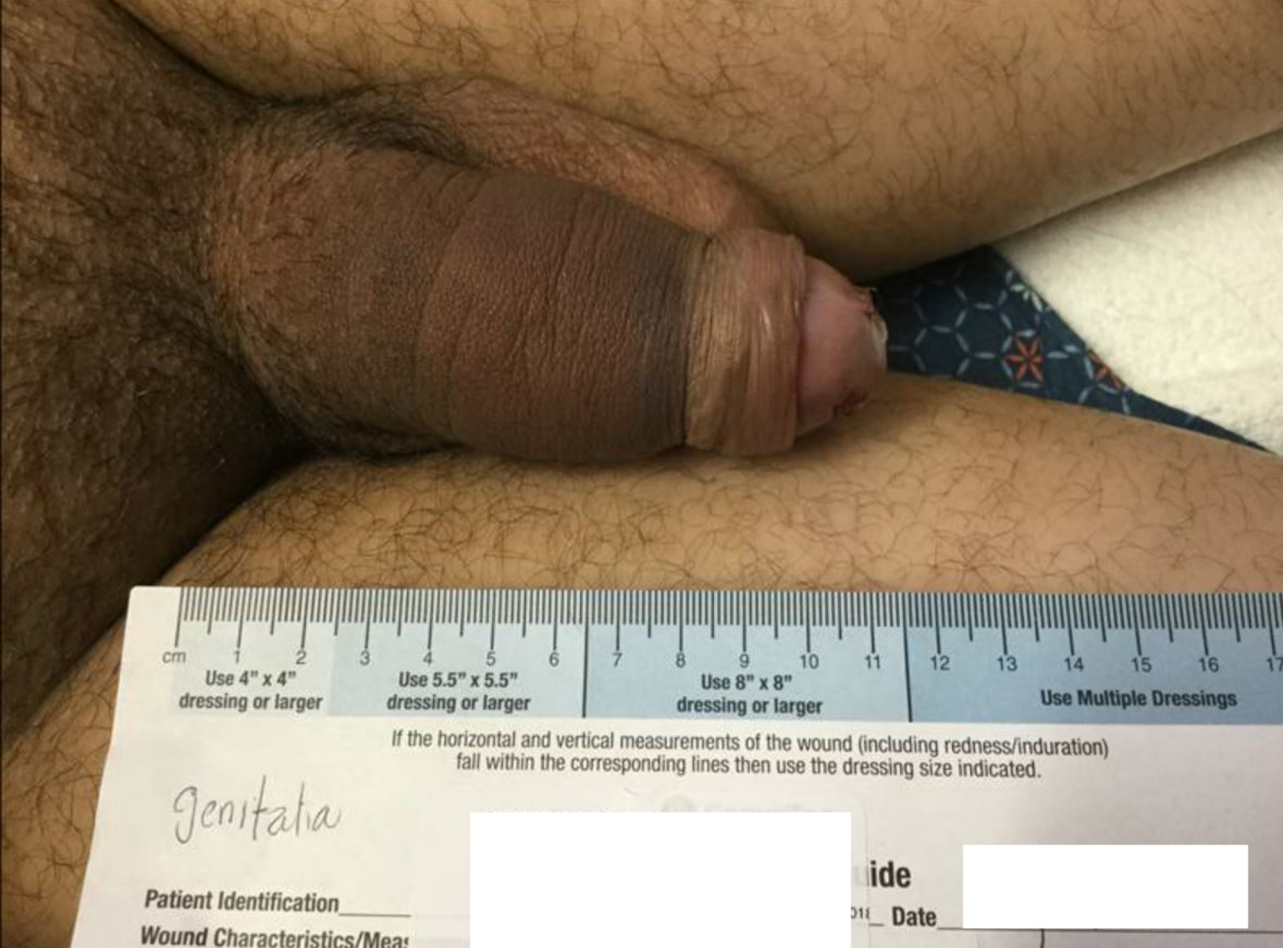
Resolution of penile erection after formation of corpora cavernosa to corpus spongiosum shunt bilaterally.

Bedside penile irrigation, aspiration, and an intracavernous phenylephrine injection were attempted again but were not helpful. The patient was again taken to the operation room where a surgical shunt was formed between corpora cavernosa and corpus spongiosum bilaterally. Postoperatively, the patient’s erection started resolving. In the next 24 hours, the patient’s priapism had resolved completely (Figure 3).

The patient received a lupron injection to decrease testosterone levels and to lower the risk of incurring priapism again in the future and was discharged with the recommendation of outpatient follow-up.

## Discussion

The term “priapism” is derived from Priapus. Priapus is the Greek god of fertility, gardening, and lust. He is usually portrayed with a massive phallus [8]. Priapism is defined as a continuous erection of the penis, which is unrelated to sexual stimulation [1], and which lasts longer than four hours in duration [2]. Priapism can occur at any age. However, in some studies, a bimodal distribution of incidence is described. The ranges given are five-ten years in children and 20-50 years in adults [9].

Three types of priapism have been identified:

I)                    Ischemic (low-flow or veno-occlusive) priapism

II)                  Nonischemic (high-flow or arterial) priapism

III)                Stuttering (recurrent or intermittent) priapism

Ischemic priapism is the most common form, and it is the cause in >95% of the cases [3-4]. It is characterized by a marked limitation of cavernous arterial flow [3]. If left untreated, ischemic priapism can lead to ischemia, necrosis, and subsequent fibrosis and erectile dysfunction of the penis [10]. Stuttering priapism is a type of ischemic priapism most commonly seen in patients suffering from sickle cell disease. It has a similar presentation to ischemic priapism, and it is characterized by recurrent episodes which usually resolve on their own but last less than three hours [11]. Those patients who do not have any underlying predisposing etiologies, like the one presented in this report, are referred to as having idiopathic stuttering priapism. The underlying pathophysiology of this condition points towards the deficiency of endothelial nitric oxide in the penis thus causing downregulation of the downstream effectors [12-13]. This causes the control of cavernous smooth muscle to operate at a lower point. Thus, any type of stimulus can induce an erectile episode for a prolonged period of time.

Priapism can be diagnosed by a range of diagnostic modalities. History and physical examination are of prime importance in this regard [14-15]. Arterial and ischemic types can be differentiated by analyzing blood gases. Ischemic priapism has pO_2_ <40 mmHg, pCO_2_ >60 mmHg, and pH <7.25. Color duplex ultrasound too has a role in the differentiation of the two types and can identify 70% of the cases of arterial priapism [16].

There are a range of options for treating stuttering priapism. These include medical as well as surgical modalities. Those cases which are prolonged and progress to the ischemic type require immediate management using corporal aspiration along with phenylephrine injections [2]. In patients suffering recurrent episodes, prophylactic treatment may be employed. These can be hormonal in nature like gonadotropin-releasing hormone (GnRH) agonists or antagonists, diethylstilbestrol, or ketoconazole. Other options that may have some effect include pseudoephedrine, digoxin, terbutaline, etilefrine, phosphodiesterase-5 (PDE5) inhibitors, and gabapentin [11]. In cases where the conservative options are exhausted and the episode does not resolve, surgical options like creating a shunt between the corpora cavernosa and glans or corpus spongiosum can be considered [11]. In the event that the patient still has priapism even after making a shunt, implantation of a penile prosthesis may help in the preservation of sexual function and penile length [5].

## Conclusions

Treatment of idiopathic stuttering priapism remains a dilemma. Medical management is not always successful. Often times, surgical treatments in the form of distal and/or proximal shunts are necessary to relieve an episode.
